# Exploring the active components and potential mechanisms of *Rosa roxburghii* Tratt in treating type 2 diabetes mellitus based on UPLC-Q-exactive Orbitrap/MS and network pharmacology

**DOI:** 10.1186/s13020-023-00713-z

**Published:** 2023-02-06

**Authors:** Chenxiao Shen, Yu Wang, Hui Zhang, Wei Li, Wenyue Chen, Mingqing Kuang, Yuelin Song, Zhangfeng Zhong

**Affiliations:** 1 Macao Centre for Research and Development in Chinese Medicine, Institute of Chinese Medical Sciences, University of Macau, Macao, SAR 999078 China; 2 Guangzhou Wanglaoji Health Industry Co, Ltd, Guangzhou, 510632 China; 3 Modern Research Center for Traditional Chinese Medicine, School of Chinese Materia Medica, Beijing University of Chinese Medicine, Beijing, 100029 China

**Keywords:** Edible and medicinal plants, *Rosa roxburghii* Tratt, Cili, Type 2 diabetes mellitus, Network pharmacology

## Abstract

**Background:**

Type 2 diabetes mellitus (T2DM) is a global disease with growing prevalence that is difficult to cure**.** *Rosa roxburghii* Tratt is an edible and medicinal plant, and modern pharmacological studies have shown that it has potential anti-diabetic activity. This is the first study to explore the active components and potential mechanisms of *Rosa roxburghii* Tratt fruit for treating T2DM based on UPLC-Q-Exactive Orbitrap/MS and network pharmacology.

**Methods:**

The active components of *Rosa roxburghii* Tratt fruit were obtained from UPLC-Q-Exactive Orbitrap/MS analysis and retrieval in the SciFinder, PubMed, Web of Science, and CNKI databases. The potential targets of the active components were obtained from the SwissTargetPrediction and PharmMapper databases. The disease targets for T2DM were obtained from GeneCards, OMIM, TTD, DisGENent, and GEO databases. The intersection of the two datasets was used to obtain the potential targets of *Rosa roxburghii* Tratt fruit against T2DM. The target protein interaction network was constructed using the String database and Cytoscape software. The R software ClusterProfiler package was used for target enrichment analysis and the Cytoscape CytoNCA plug-in was used to screen core targets. Molecular docking and result visualization were performed using PyMOL and Autodock Vina software.

**Results:**

We obtained 20 bioactive ingredients, including alphitolic acid, quercetin, and ellagic acid, as well as 13 core targets, such as AKT1, TNF, SRC, and VEGFA. All bioactive ingredients in *Rosa roxburghii* Tratt fruit were active against T2DM-related therapeutic targets. *Rosa roxburghii* Tratt fruit may play a therapeutic role in T2DM by regulating the PI3K/AKT, RAS, AGE-RAGE, and other signaling pathways.

**Conclusions:**

This study explored the active components and potential mechanisms of *Rosa roxburghii* Tratt fruit in the treatment of T2DM, laying the foundation for a further experimental study based on pharmacodynamic substances and their mechanisms of action.

**Graphical Abstract:**

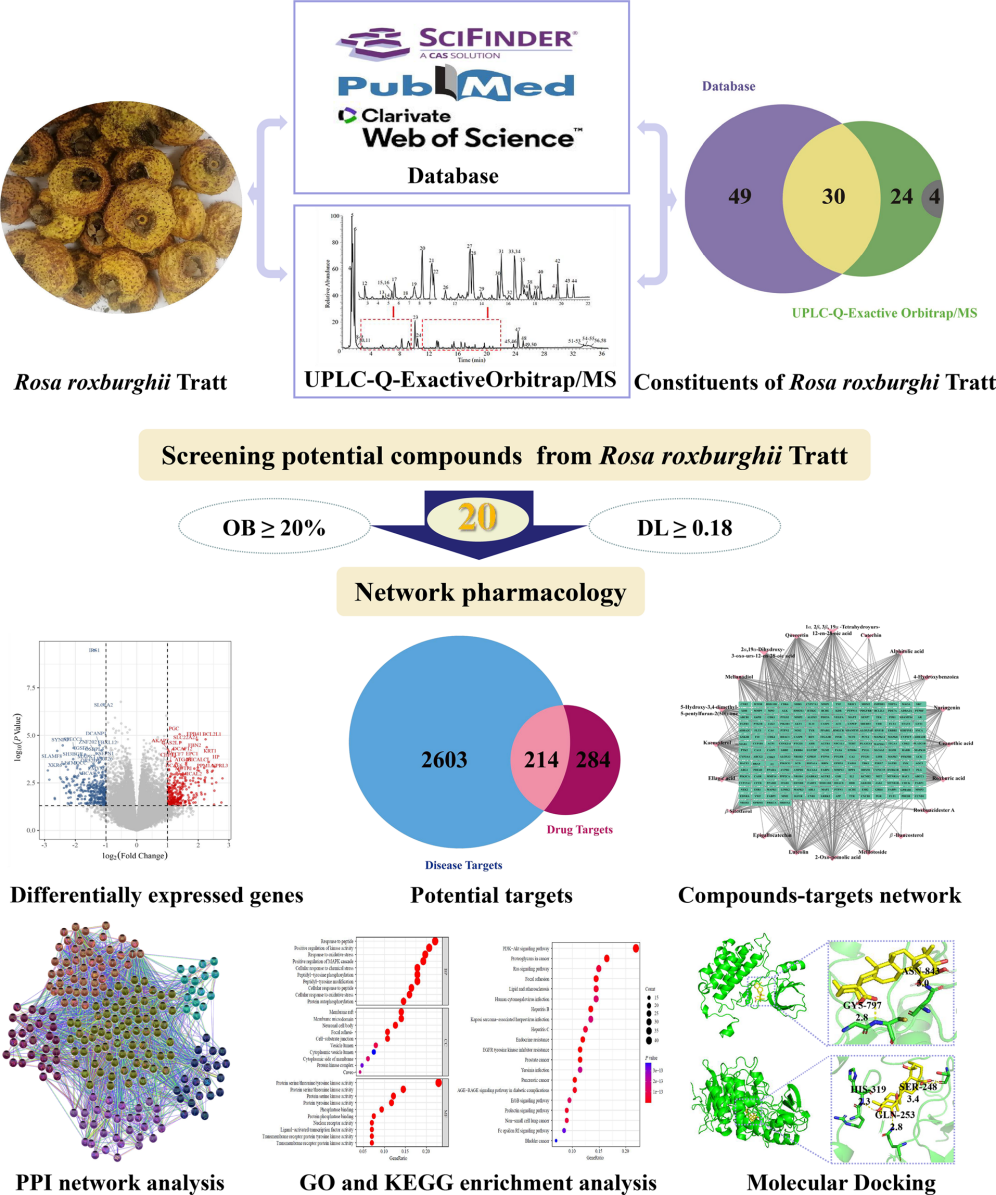

**Supplementary Information:**

The online version contains supplementary material available at 10.1186/s13020-023-00713-z.

## Introduction

Type 2 diabetes mellitus(T2DM), a critical risk factor for cerebral infarction, cardiovascular disease, blindness, and kidney failure, is caused by the joint influence of lifestyle and genetic components [1]. At present, synthetic anti-diabetic agents are widely used in clinical settings for T2DM treatment but have been reported with many side effects [2]. Natural products are abundant sources for drug discovery [3, 4]. In recent years, a large number of edible and medicinal plants have received widespread attention particularly given the few side effects it carries and their multi-target treatments.

*Rosa roxburghii* Tratt (Family: Rosaceae) is a shrub indigenous to Guizhou province in southwest China. It is an edible and medicinal plant named “Cili” in Chinese. The initial record of this plant can be traced back to the ancient book “Gui Zhou Tong Zhi” (Qing Dynasty, 1697 A.D) [5]. *Rosa roxburghii* Tratt fruit has been always used as medicinal product in the form of expressed juice to maintain the hydrophobic components and contains a variety of biologically active metabolites such as triterpenes, flavonoids, and rich nutrients such as trace elements, vitamins, polysaccharides, dietary fibers, unsaturated fatty acids, and superoxide dismutase [6–11]. Modern pharmacological research shows that *Rosa roxburghii* Tratt exhibits many pharmacological effects, including antitumor activity, anti-diabetic activity, anti-inflammatory activity, anti-apoptosis activity, anti-radiation activity, and antioxidant activity [12–18].

Network pharmacology is a new discipline based on high-throughput omics data analysis, computer virtual computing, network database retrieval, and on the theory of system biology, which conducts network analysis on biological systems, predicts drug targets as a whole, and improves drug discovery efficiency [19]. At present, it has been widely used to screen active ingredients, explore drug action mechanisms, and study disease pathogenesis. Despite the growing research that has demonstrated the anti-diabetic activity of *Rosa roxburghii* Tratt, the characterization of its effective compounds and the exact mechanisms of action have not been systemically demonstrated [20, 21]. In this study, we first used bioinformatics analysis including network pharmacology and molecular docking to explore the mechanisms of *Rosa roxburghii* Tratt fruit in T2DM treatment.

## Materials and methods

### Active ingredients dataset

We searched the chemical constituents of *Rosa roxburghii* Tratt fruit from the literature in the SciFinder, PubMed, Web of Science, and CNKI databases. To ensure the accuracy of the components, we characterized the chemical components of *Rosa roxburghii* Tratt fruit by UPLC-Q-Exactive Orbitrap/MS. Subsequently, the comprehensive classification of drug similarity and pharmacokinetic properties of the searched compounds were evaluated according to the ADMET (absorption, distribution, metabolism, excretion, and toxicity) guidelines. Through the traditional Chinese medicine system pharmacology analysis platform TCMSP (http://tcmspw.com/tcmsp.php) and ADMETlab 2.0 (https://admetmesh.scbdd.com/) platform, we selected candidate drugs that fulfilled the criteria of oral bioavailability (OB) ≥ 20% and drug similarity (DL) ≥ 0.18 [22, 23].

### Characterization of the chemical profile of *Rosa roxburghii* Tratt fruit using LC − MS/MS

Fifteen batches of fresh fruits from *Rosa roxburghii* Tratt were collected from Longli County, Qiannan City, Guizhou Province, China. Authentic compounds, including catechin, epicatechin and proanthocyanidin B2, were prepared from our laboratory. The purity of each collected compound was determined to be greater than 98% by LC-MS/MS. LC-MS grade acetonitrile (ACN), methanol, and formic acid were supplied by Thermo-Fisher (Pittsburgh, PA). Deionized water was prepared from a Milli-Q Integral Water Purification System (Bedford, MA).

The fresh fruits were washed and dried in an oven at 50 °C for 72 h. The dry fruit was deseeded and individually pulverized into powders. Approximately 50 mg powders were sampled from each batch and extracted with 70 volumes (g/mL) of methanol and ultrasonicated for 30 min. After centrifugation for 10 min, the supernatants were filtered through a 0.22 μm Nylon membrane filter to produce fifteen extracts. Furthermore, 50 μL aliquots from all batches of extracts were sampled and pooled to produce a homogenized solution, and the mixture served as a quality control (QC) sample for subsequent analyses.

UPLC-Q-Exactive Orbitrap/MS (Thermo-Fisher Scientific, Waltham, MA) was performed on analytical measurements. Chromatographic separation was achieved on the Waters HSS T3 column (2.1 mm × 100 mm). The mobile phase consisted of 0.1% formic acid water (A) and ACN (B) and was programmed as follows: 0–10 min, 6–12% B; 10–18 min, 12–30% B; 18–26 min, 30–36% B; 26–31 min, 36–40% B, 31–32 min, 40–95% B, 32–33 min, 95–6% B; 33–37 min, 6% B; and flow rate, 0.2 mL/min. The injection volume was set at 2 μL, and the column oven was maintained at 35 °C. The full MS^1^ scan had 70 000 FWHM resolution between *m/z* 70–1050, and the auto-triggered MS/MS acquisition was conducted at 175 000 FWHM resolution within* m/z *70–1050 through a data-dependent top 10 acquisition algorithm. The isolation width of the precursor ion was 1.5 Da, the collision energy was set as 30, 50, and 70 eV for the positive and negative ESI mode. The heated capillary temperature was set at 350 °C. The nitrogen sheath gas was set at 35 arbitrary units and the auxiliary gas at 10 arbitrary units. The capillary sprayer voltages were fixed at 3800 V and − 2800 V for positive and negative polarities, respectively. Xcalibur software (Version 2.1.0, ThermoFisher Scientific) was utilized for data processing.

### Potential drug targets of *Rosa roxburghii* Tratt fruit

The compounds were introduced into the SwissTarget Prediction database [24] (http://www.swisstargetprediction.ch/) and the PharmMapper Server [25] (http://lilab-ecust.cn/pharmmapper/) to predict potential targets. The target proteins with “popular organisms” were limited to humans. We adopted the UniProt database [26] (http://www.uniprot.org/uniprot/) to standardize target names to gene symbols.

### Identification of T2DM-related therapeutic targets

With 'type 2 diabetes mellitus' as keyword, known differentially expressed targets related to T2DM were acquired from the GEO database (https://www.ncbi.nlm.nih.gov/geo/, Series: GSE26168, GSE41762, and GSE166502). The 'GEOquery' and 'Limma' packages (https://www.bioconductor.org/) of Rmur4.2.0 were used to perform a joint analysis of multiple chips and correct data batches. Differential genes in healthy people and patients with T2DM with a *P* value < 0.05 and log2 (fold change) > 1 or log2 (fold change) < –1 were considered target genes. The targets for T2DM disease were retrieved from the following databases, Gene Cards [27] (https://www.genecards.org/), TTD [28] (https://db.idrblab.org/ttd/), DisGENent [29] (https://www.disgenet.org/web/DisGeNET/menu/home), and OMIM [30] (https://omim.org) using the keyword 'type 2 diabetes mellitus'. After removing duplicates, the identified targets from the four above databases and GEO microarrays were included in the pool of T2DM-related therapeutic targets.

### Compound-target network construction

The compound-target network was constructed using Cytoscape v3.9.0 software to visualize the graphical relationship between the active compounds in *Rosa roxburghii* Tratt fruit and the targets. The nodes of the network represent molecules from *Rosa roxburghii* Tratt fruit or the interactive targets, and the edges indicate the interactions between compounds and targets.

### PPI network construction and topological analysis

The PPI network was established using the STRING platform (https://string-db.org/). Topological analysis was carried out using the Cytoscape CytoNCA plug-in to calculate the parameters of degree centrality (DC), betweenness centrality (BC), and closeness centrality (CC), which are the most important parameters for measuring the criticality of a node in the network, and also used as the important index for the discovery of new drugs and target prediction [31–34].

### GO and KEGG enrichment analysis

The GO enrichment analysis was performed from biological process (BP), cellular component (CC), and molecular function (MF). The top 10 entries of each component were analyzed based on the *P-*value. KEGG enrichment was performed to annotate the signaling pathways involved in these targets, and the top 20 entries were selected based on the *P-value.*

### Molecular docking

Core targets were downloaded through the PDB database (https://www.rcsb.org/) to obtain protein crystal structures. The 3D structures of the compounds were constructed by ChemBio3D Ultra 14.0 software and calculated the minimum free energy. AutoDockTools 1.5.6 software was used to pretreat the proteins and compounds. We used AutoDock Vina 1.1.2 for batch docking. Molecular docking results were visualized with PyMol 4.6.0 software.

## Results

### Identification of compounds in* Rosa roxburghii* Tratt fruit

We obtained 79 chemical constituents of *Rosa roxburghii* Tratt fruit by literature search, including 23 triterpenoids, 14 flavonoids, 17 tannins, 3 steroids, 7 phenolic acids, 7 organic acids and fatty acids, and 8 other compounds. We then characterized the chemical components of *Rosa roxburghii* Tratt fruit by UPLC-Q-Exactive Orbitrap/MS.

**Fig. 1:**
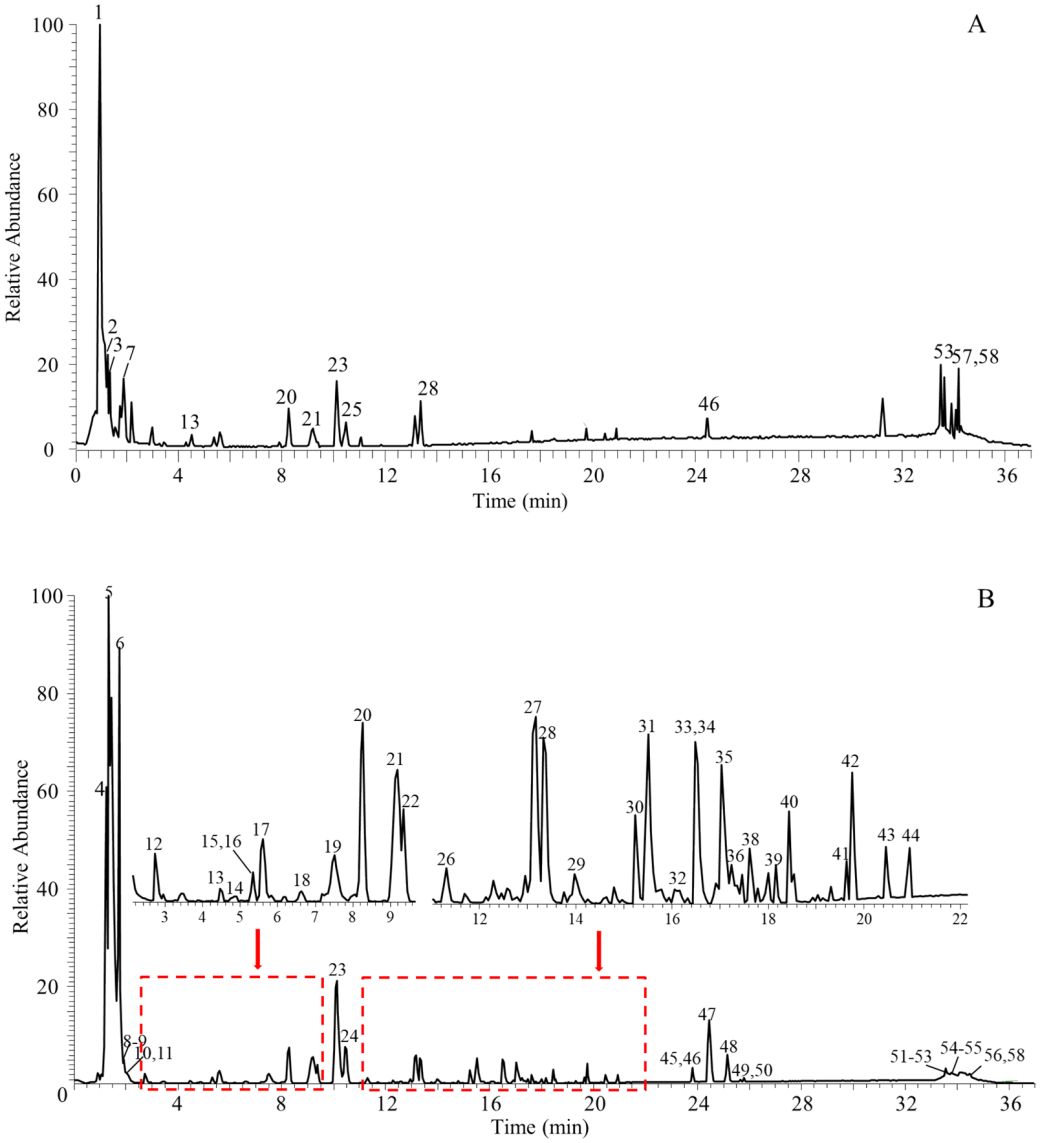
Total ion chromatograms of the QC sample of Rosa roxburghii Tratt fruit using UPLC-Q-Exactive Orbitrap/MS. **A**: positive ion mode. **B**: negative ion mode

The QC sample was measured by UPLC-Q-Exactive Orbitrap/MS. The base peak current chromatogram (BPC) is shown in Fig. 1. After in-depth data processing, a total of 58 compounds were captured and 54 components were tentatively identified, including 19 triterpenoids, 17 flavonoids, 7 organic acids, 3 amino acids, 5 tannins, 2 phenolic acids, and 1 coumarin (Additional file 1: Table S1), by carefully queries of accessible databases (*e.g.*, PubChem, HMDB, ChemSpider, MyCompoundID, Massbank.) and applying those proposed mass fragmentation pathways [8, 35, 36]

Seven compound families were identified: triterpenoids, flavonoids, organic acids, amino acids, tannins, phenolic acids, and coumarins. Among them, triterpenoids and flavonoids were the primary chemical clusters for structural annotation. For triterpenoids, the frequently observed mass fragmentation pathways included cleavage of the glucosyl residue (162.05 Da), neutral losses of HCOOH (46.00 Da), H_2_O (18.01 Da), CO (27.99 Da), and CO_2_ (43.98 Da). Taking **58** for example, the compound generated the deprotonated molecular ion at *m/z* 471.34 ([M − H]^−^, C_30_H_58_O_10_), and observed a set of fragment ion species (Additional file 1: Figure S1) at *m/z* 427.35 ([M − H − HCOOH]^−^) and 453.33 ([M − H − H_2_O]^−^). Compound **58** was tentatively identified as alphitolic acid, and the mass fragmentation pathway corresponding to those fragment ions is proposed in Additional file 1: Figure S2. In a similar pattern, the structural annotation was performed for the other 17 triterpenoids (41–57).

Regarding flavonoids, those featured fragment ions such as ^1,2^A^−^, ^1,2^B^−^, ^1,3^A^−^, ^1,3^B^−^, ^1,4^A^−^, ^1,4^B^−^, ^0,4^A^−^, and ^0,4^B^−^ that were termed by following the ion nomenclature rule were observed frequently. There were also other neutral losses of H_2_O (18.01 Da), CO (27.99 Da), and CO_2_ (43.98 Da) according to the relative literature [34]. For instance, compound 23, the MS/MS spectrum (Additional file 1: Figure S3) of its deprotonated molecular ion (*m/z* 289.07 [M − H]^−^, C_15_H_14_O_6_) yielded fragment ions at *m/z* 245.08 ([M − H − CO_2_]^−^), 203.05 ([M − H − CO_2_ − C_2_H_2_O]^−^), 271.06 ([M − H − H_2_O]^−^), 179.03 ([M − H − C_6_H_2_O_2_]^−^), 163.04 ([M − H − H_2_O − C_6_H_2_O_2_]^−^), 151.04 ([M − H − C_7_H_5_O_3_]^−^), 137.02 ([M − H − C_8_H_8_O_3_]^−^), 123.04 ([M − H − C_7_H_5_O_3_ − CO]^−^), 109.03 ([M − H − C_9_H_8_O_4_]^−^) [37]. Furthermore, the proposed mass fragment ions are depicted in Additional file 1: Figure S4. As expected, the cleavage of the RDA reaction and neutral losses of CO, CO_2_, and H_2_O served as the main fragmentation channels. Therefore, compound **23** was tentatively identified as catechin by matching chromatographic and MS/MS information with an authentic compound. Similarly, the other flavonoids (**19** − **21**, **23**, **24**, **27** − **29**, **31**, **36** − **40**) were structurally annotated by applying the proposed mass cracking rules to their MS/MS spectra, and the plausible structures are summarized in Additional file 1: Table S1.

For organic acids, amino acids, tannins, phenolic acids, and coumarin, a similar program was performed on tentative identification with relative literature [8, 35, 36, 38]. Although significant efforts were made, four compounds could not yet be characterized and were thus assigned as unknown compounds.

**Fig. 2:**
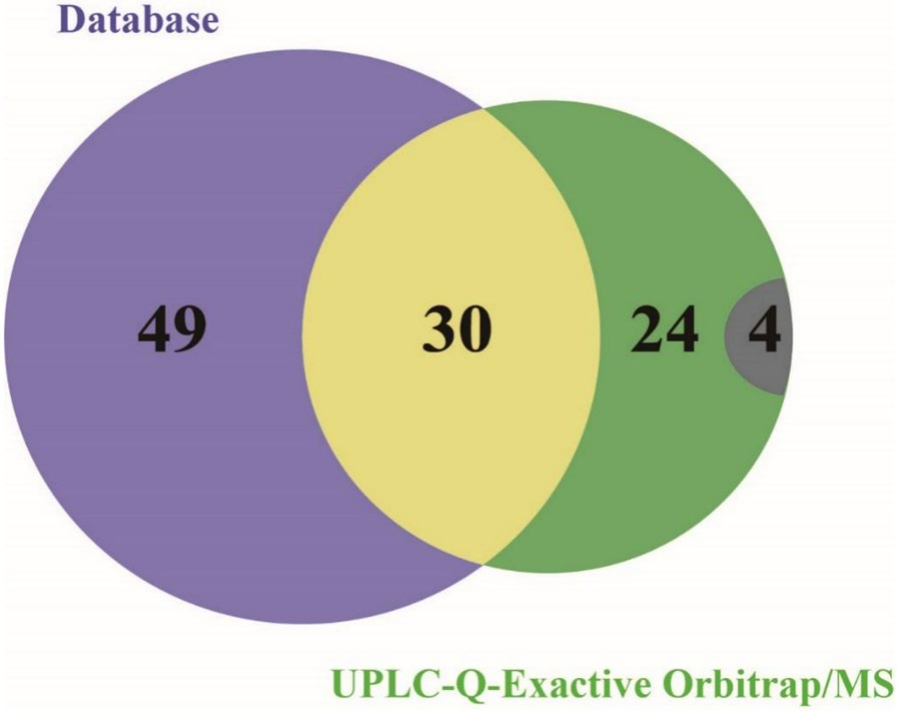
Chemical constituents of Rosa roxburghii Tratt fruit. Purple and green represent differential compounds in the UPLC-Q-Exactive Orbitrap/MS database, while the cross section represents the same compounds. The grey represents unknown compounds

We analyzed the results obtained from the database and UPLC-Q-Exactive Orbitrap/MS. Through UPLC-Q-Exactive Orbitrap/MS component identification, we obtained 30 components identical to those reported in the literature (Fig. 2). To ensure the integrity of the data, we fused the two methods to obtain 103 compounds. We used the 103 compounds as the material database of *Rosa roxburghii* Tratt fruit for further screening.

### Active ingredients and targets

**Fig. 3:**
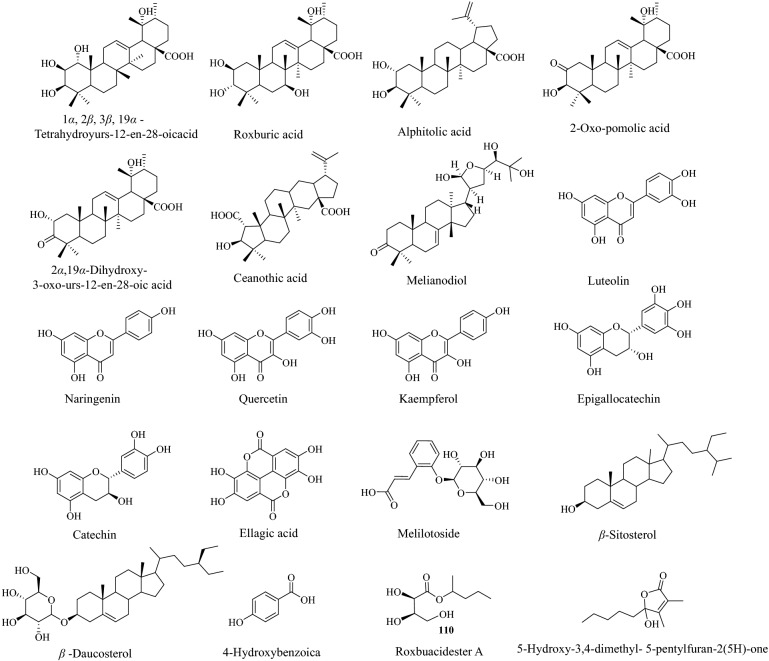
Chemical structures of the active ingredients in Rosa roxburghii Tratt fruit. The key criteria for active ingredients were oral bioavailability (OB) ≥ 20% and drug similarity (DL) ≥ 0.18

Twenty candidates (Fig. 3) were selected using the ADMET criterion with oral bioavailability (OB) ≥ 20% and drug-likeness (DL) ≥ 0.18. We then imported the 20 compounds into the SwissTarget Prediction and PharmMapper Server database for target prediction, and 498 potential targets were interactively predicted with the active molecules of *Rosa roxburghii* Tratt fruit.

### T2DM-related targets

**Fig. 4:**
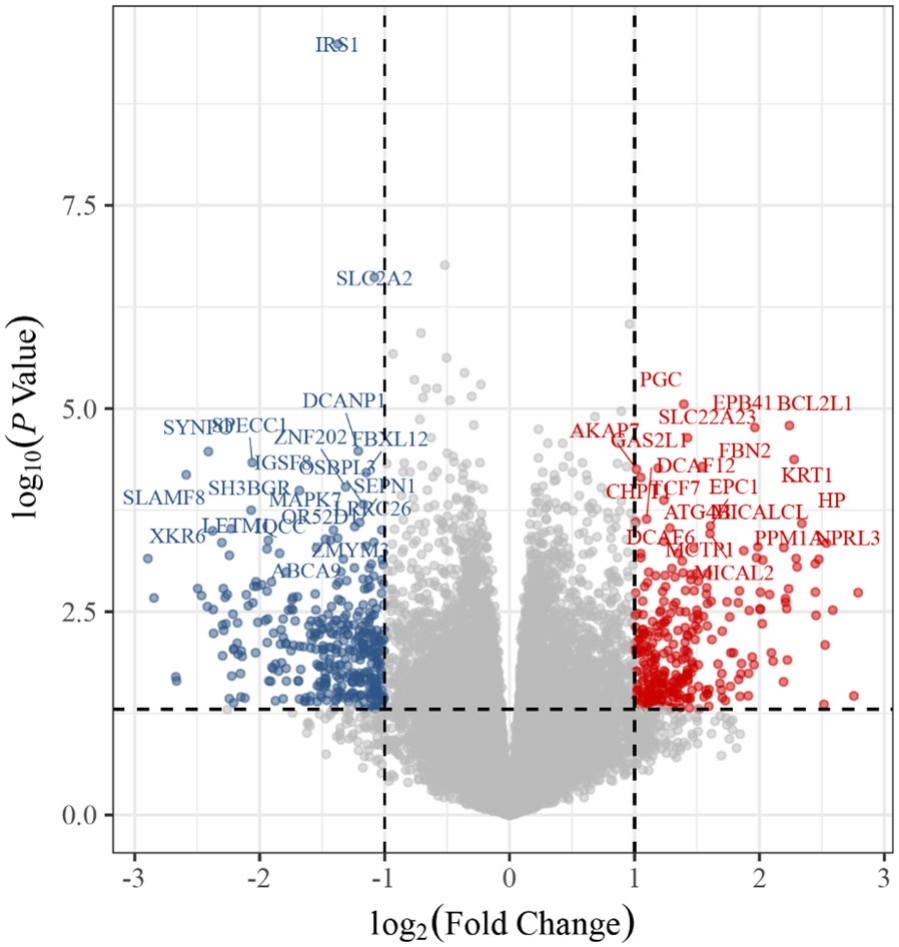
Volcano plot of differentially expressed genes in the GEO probe matrix. The red represents up-regulated genes, blue represents down-regulated genes, and gray represents not-significant genes

In the conjoint analysis of the GEO microarray GSE26168, GSE41762, and GSE166502 in the GEO database with the criteria of *P*-value < 0.05 and log2 (fold change) > 1 or log2 (fold change) <  − 1, we identified 661 differentially expressed genes associated with T2DM. Among them, there were 299 up-regulated genes and 362 down-regulated genes that were used to construct volcano maps (Fig. 4). Furthermore, we integrated disease targets from GeneCards, DisGeNET, TTD, OMIM, and GEO databases to the final identification of 2817 disease targets after removing duplicates.

### Drug targets-disease targets network of *Rosa roxburghii* Tratt fruit

**Fig. 5:**
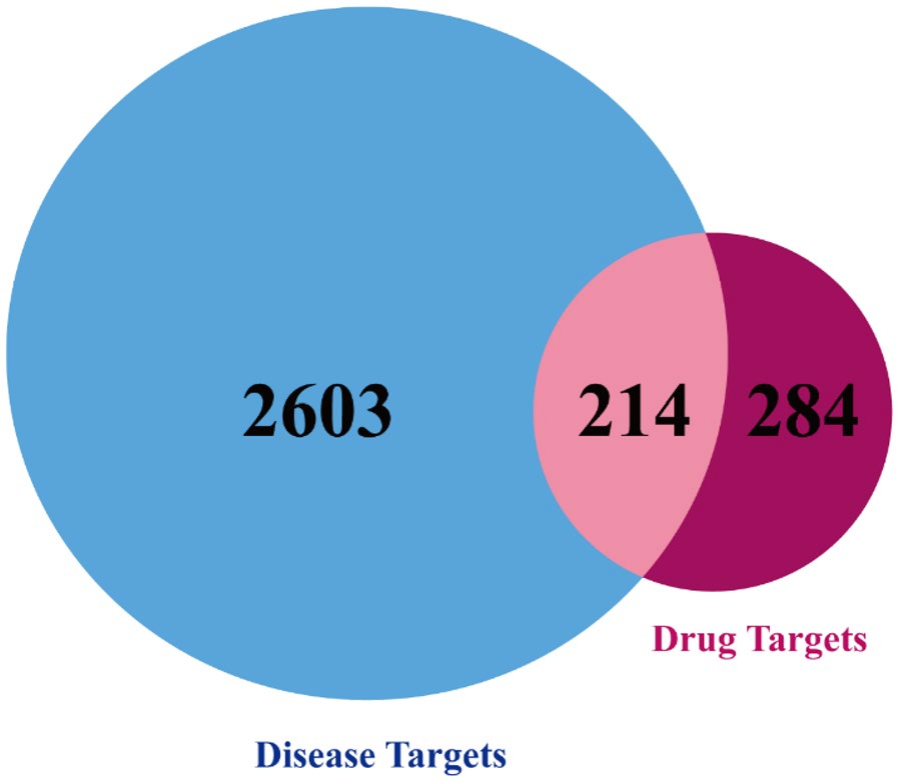
Venn diagram of the targets in drug and disease. The blue represents the T2DM targets of Rosa roxburghii Tratt fruit, the red represents compound targets, and the intersection represents the potential target of *Rosa roxburghii* Tratt fruit for the treatment of T2DM

**Fig. 6:**
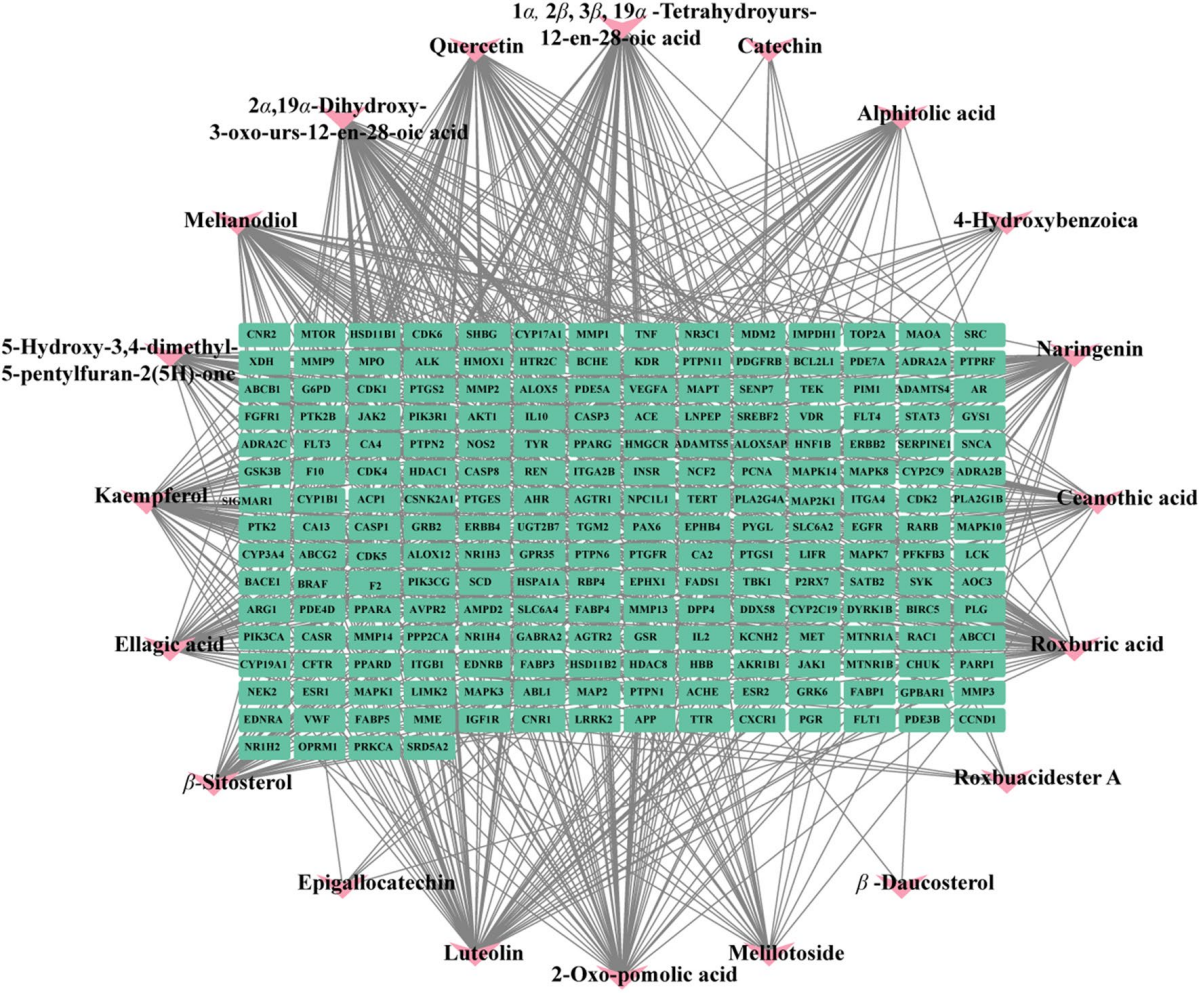
Compound-target network of Rosa roxburghii Tratt in treating T2DM. The green rectangle nodes represent the T2DM target proteins and the orange ellipse nodes represent compounds

The drug target genes intersected with the T2DM disease gene and were plotted on a Venn diagram (Fig. 5). The total number of their intersected target genes was 214. Figure 6 shows the compound-target network with 234 nodes and 783 edges included, the green rectangle nodes represent the T2DM target proteins, and the yellow triangle node represents the compounds.

### PPI network construction and core target protein screening

**Fig. 7:**
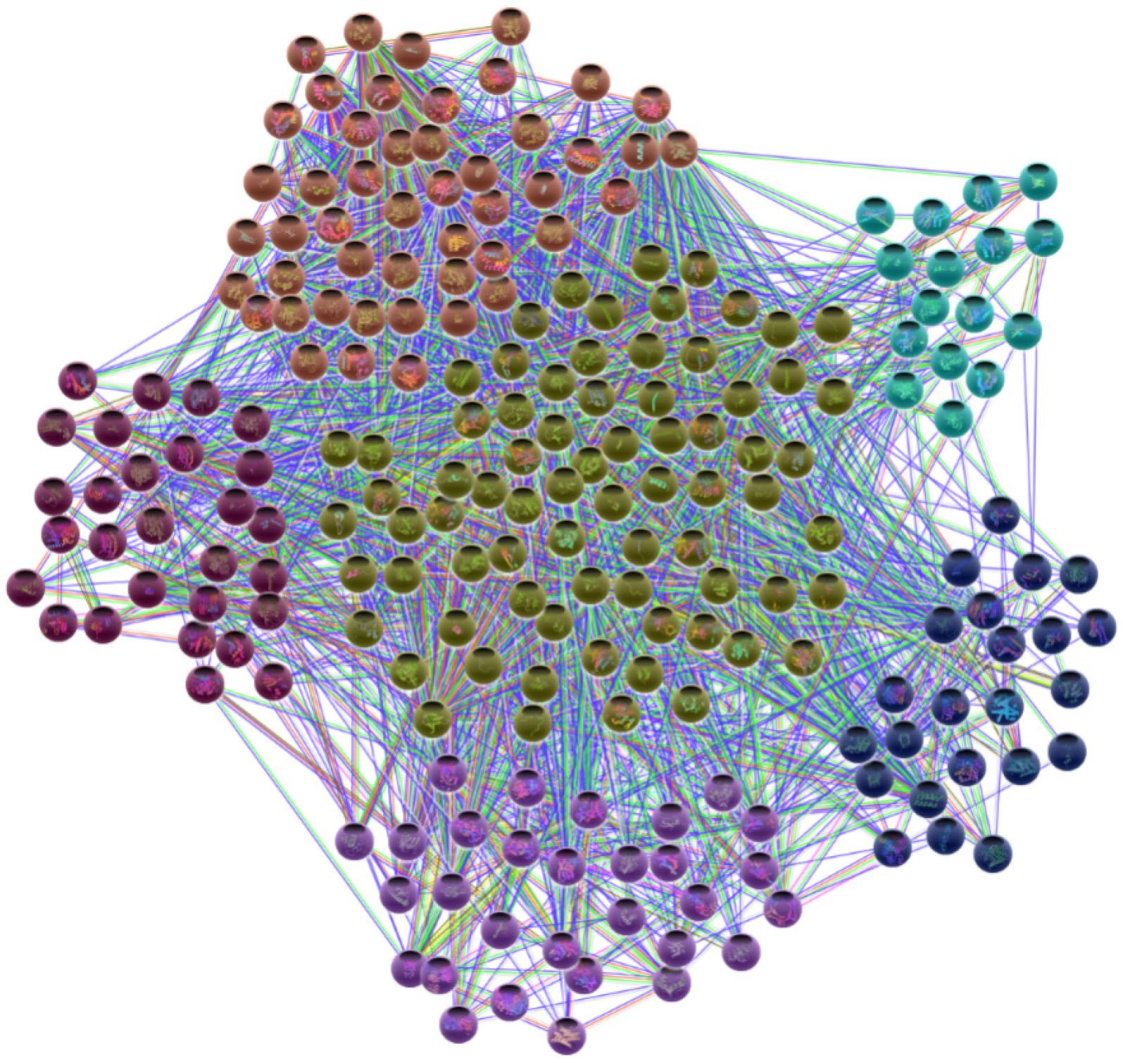
PPI network construction and visualization analysis of 214 nodes and 3007 edges. Nodes represent overlapping target proteins, and edges between nodes represent the target-target interaction

The intersection targets were imported into the STRING database with hidden targets without any interactions. We then imported the PPI data into Cytoscape to draw the PPI network in Fig. 7. We selected targets larger than the median of DC, BC, and CC, repeated the same method three times, and selected the target as the core target, with the cutoff values of DC > 76, BC > 951.55, and CC > 0.59. There were 13 target proteins identified as the core target proteins (Table 1).

**Table 1:** Core targets of *Rosa roxburghii* Tratt fruit in the treatment of T2DM

Number	Core targets	Degree	Betweenness	Closeness
1	AKT1	128	4132.90	0.71
2	TNF	124	3789.15	0.69
3	SRC	108	1936.38	0.66
4	VEGFA	105	1366.52	0.65
5	EGFR	98	1590.71	0.64
6	CASP3	97	1146.73	0.64
7	MAPK3	96	1249.94	0.63
8	STAT3	94	1136.86	0.63
9	PPARG	85	2597.99	0.61
10	ESR1	83	1270.22	0.60
11	mTOR	81	980.66	0.61
12	PTGS2	79	1135.57	0.60
13	MAPK1	78	1111.02	0.60

*AKT1* serine/threonine kinase 1, *TNF* tumor necrosis factor, *SRC* steroid receptor coactivator, *VEGFA* vascular endothelial growth factor A, *EGFR* epidermal growth factor receptor, *CASP3* caspase-3, *MAPK3* mitogen-activated protein 3, *STAT3* signal transducer and activator of transcription 3, *PPARG* peroxisome proliferator-activated receptor gamma, *ESR1* estrogen receptor 1, *mTOR* mammalian target of rapamycin, *PTGS2* prostaglandin-endoperoxide synthase 2, *MAPK1* mitogen-activated protein kinase 1

### GO and KEGG analyses

**Fig. 8:**
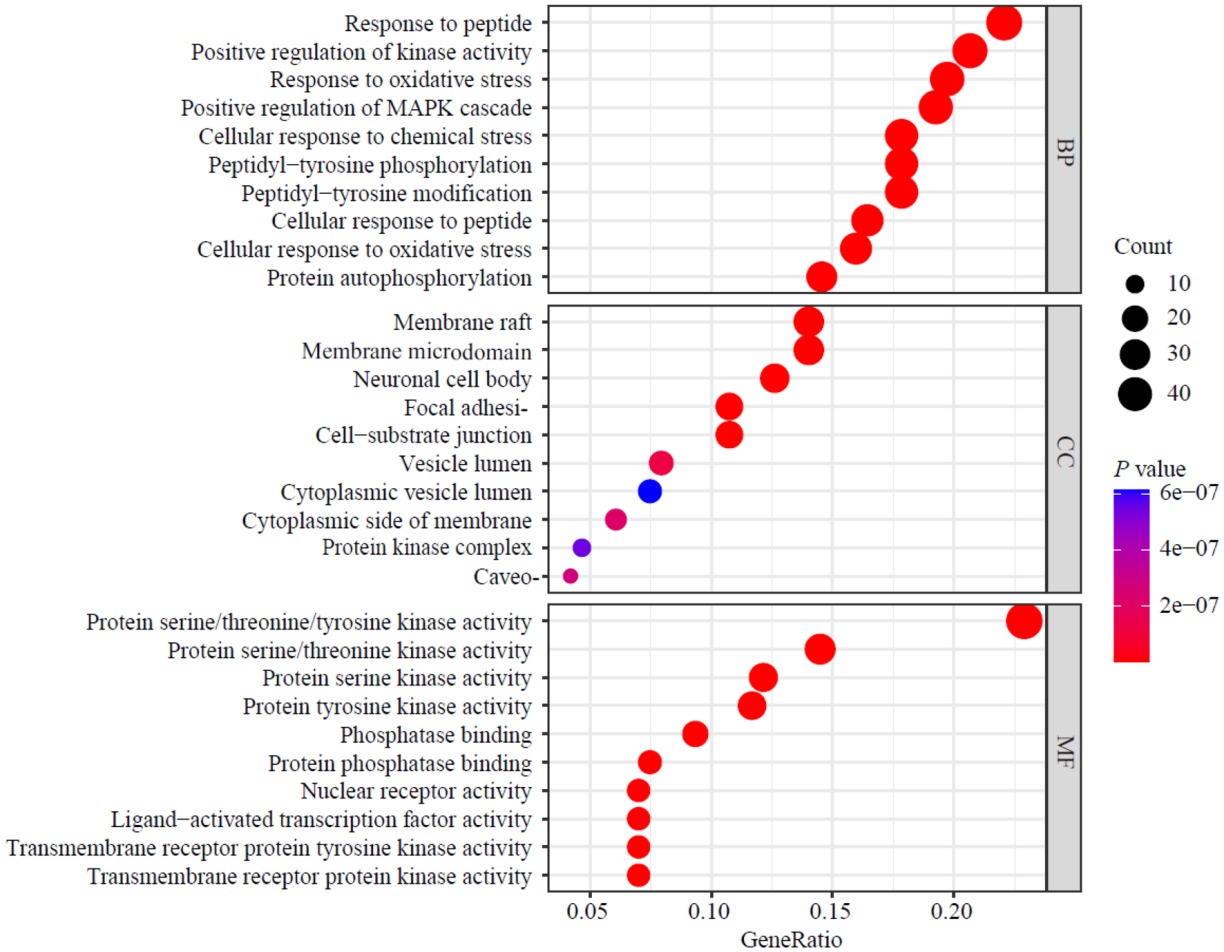
GO enrichment analysis. The top 10 terms with a *P* value < 0.05 were selected. The size of the dots represents the number of genes, and the color represents the significance of the *P* value

**Fig. 9:**
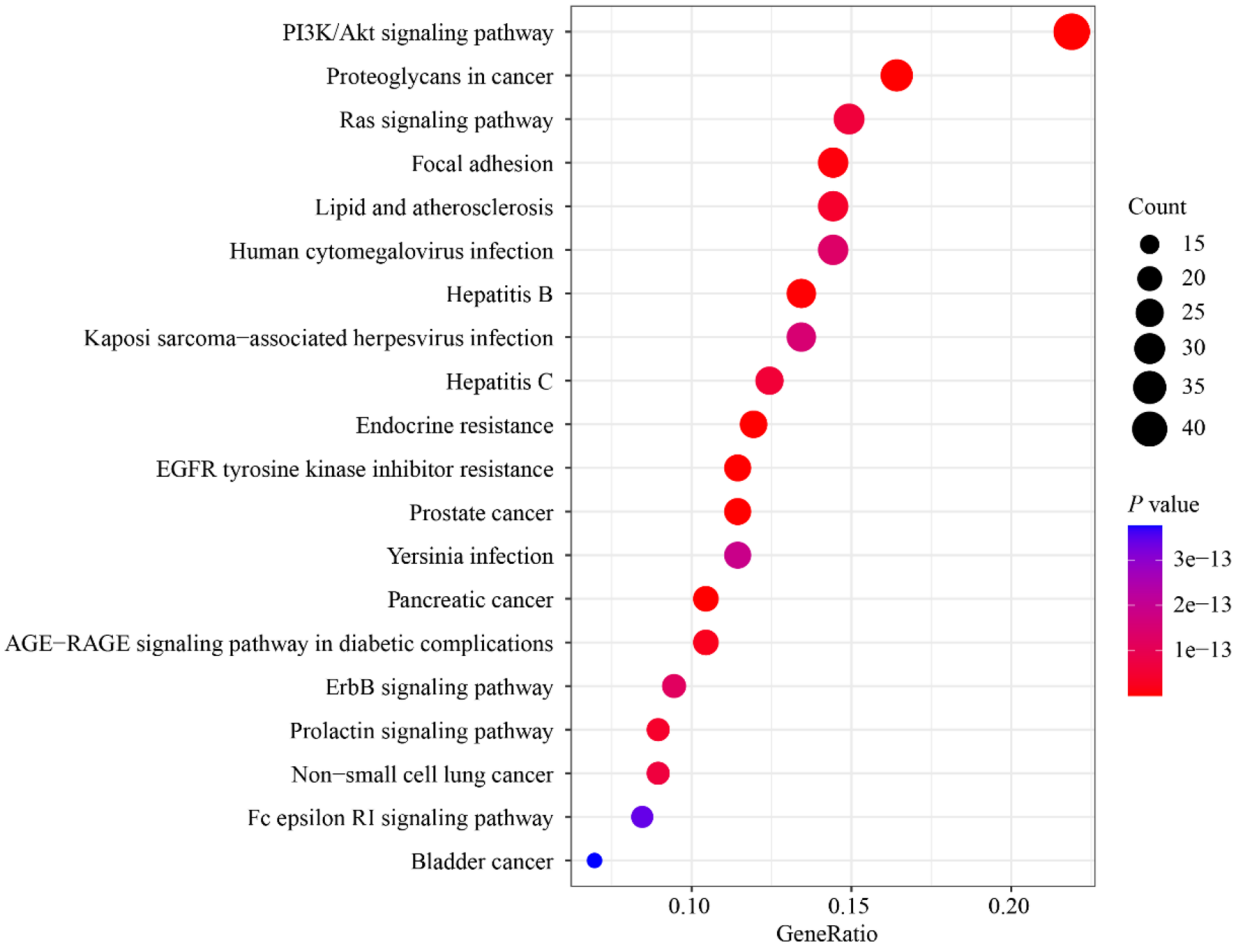
KEGG pathway enrichment analysis. The top 20 significant pathways with a *P* value < 0.05 were identified. The size of the dots represents the number of genes, and the color represents the significance of the *P* value

There were 2643 enrichment GO terms with a *P* value < 0.05, including 2370 in biological processes, 93 in a cellular component, and 180 in molecular function. The top 10 terms among the enrich entries are shown in Fig. 8. Through the analysis of the enriched GO items, these genes distributed in biological processes, cellular components, and molecular functions were manifested primarily in response to peptide, regulation of protein serine/threonine/tyrosine kinase activity, response to oxidative stress, cell membrane, and protein kinase regulator activity. Similarly, we also performed a KEGG analysis to determine which pathways exert significant effects on pathological mechanisms. As shown in Fig. 9, the 20 most significantly enriched pathways (*P*-value < 0.05), which identified, the PI3K (phosphatidylinositol 3-kinase)/AKT (serine/threonine kinase) signaling pathway, cancers, viral infection, and AGE-RAGE signaling pathway in diabetic complications.

### Molecular docking

**Fig. 10:**
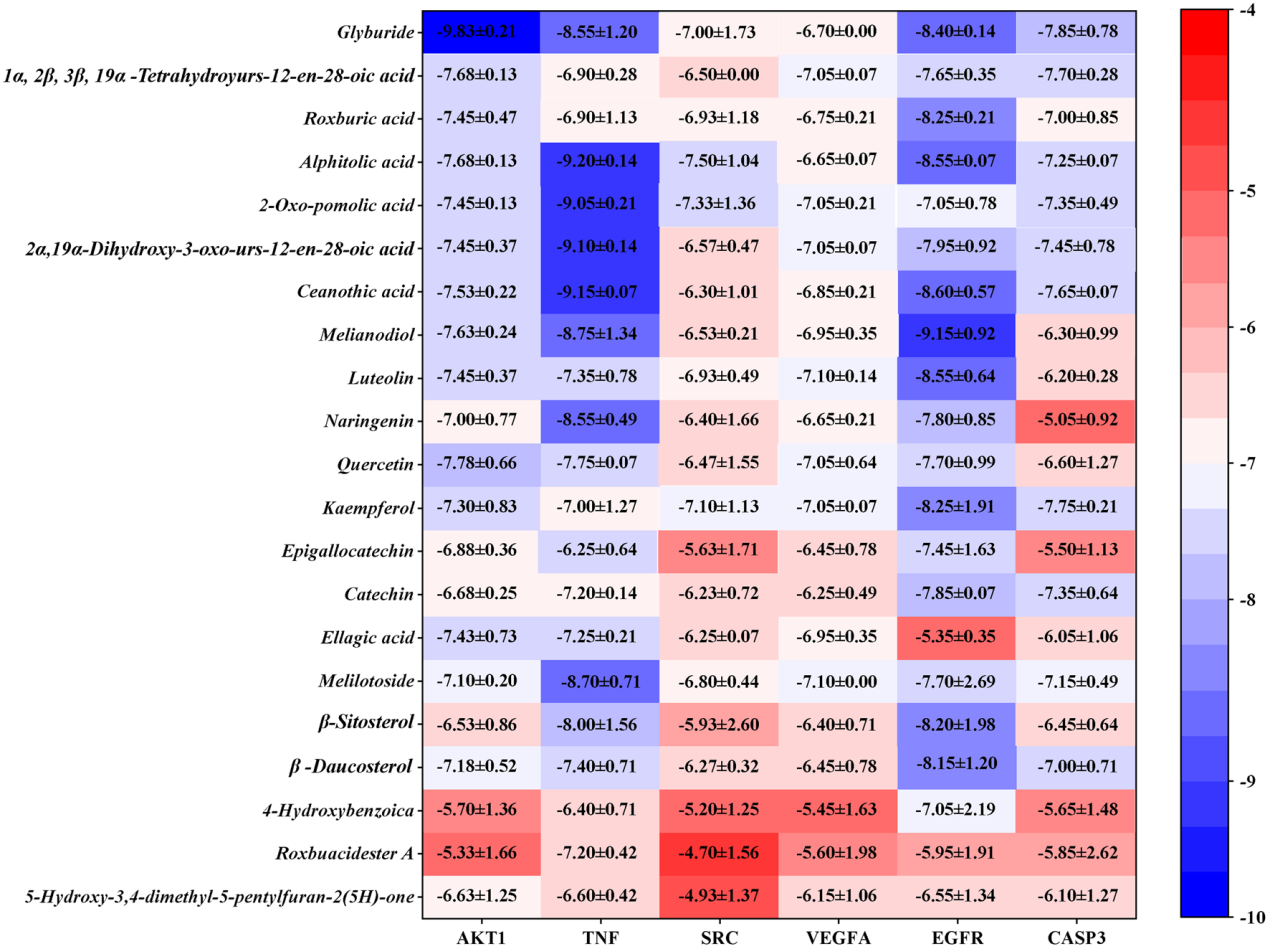
Binding energies of 20 key compounds to 6 core target proteins, with glyburide as the control drug. The ordinate represents the top six core targets, the abscissa represents the core compound, and the numerical value is the docking energy (Mean ± SD, N = 3)

**Fig. 11:**
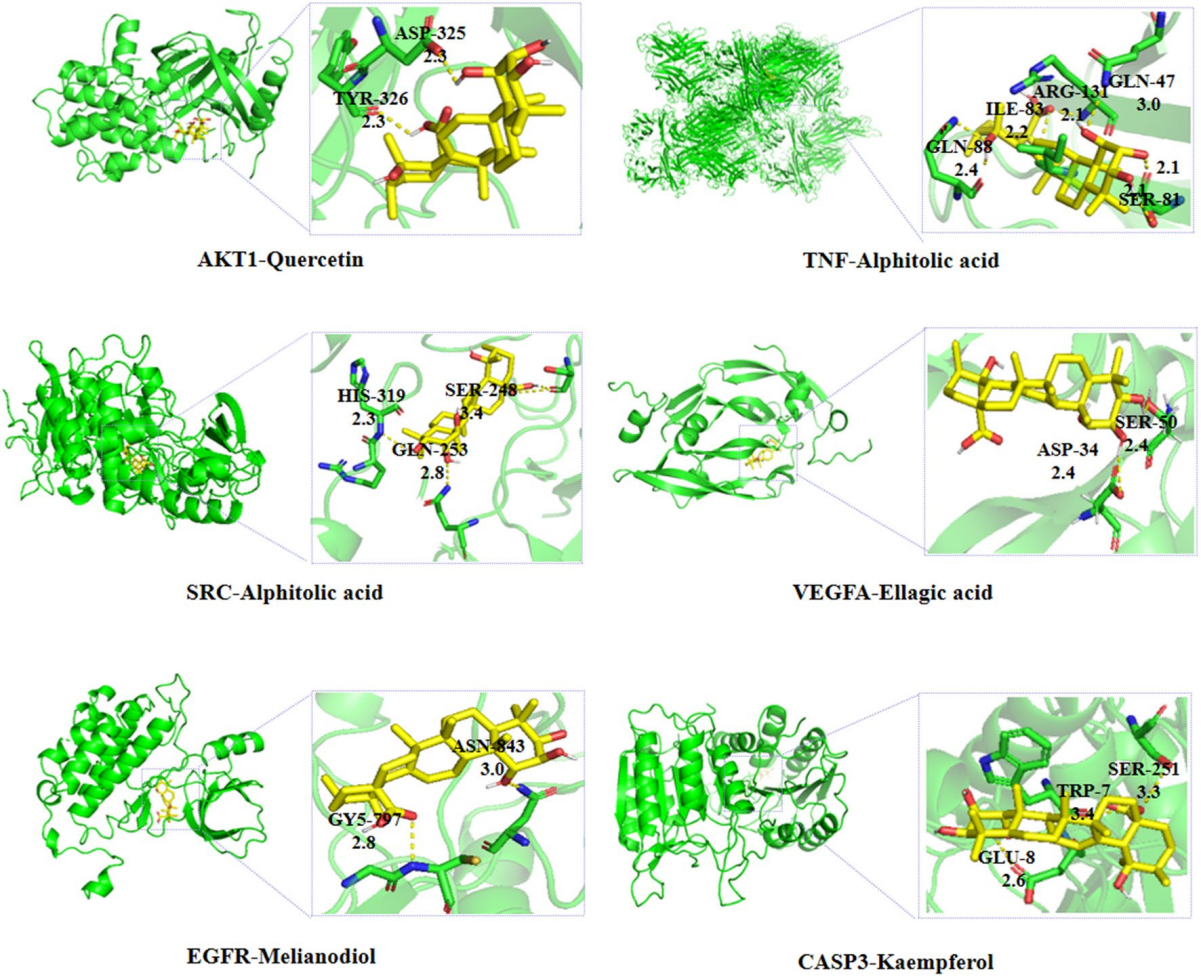
Visualization of molecular docking of the key target active ingredient. Green represents the receptor proteins, yellow represents the ligand compounds, and the label is the binding site and the distance

We selected the first six target proteins (AKT1: serine/threonine kinase 1; TNF: tumor necrosis factor; SRC: steroid receptor coactivator; VEGFA: vascular endothelial growth factor A; EGFR: epidermal growth factor receptor; CASP3: caspase-3) as receptor proteins for molecular docking with corresponding compounds and Glyburide as the control drug. The results of molecular docking are shown on the heat map (Fig. 10). The top-ranked docking results for each target are shown in Fig. 11.

## Discussion

T2DM is one of the most prevalent and serious metabolic diseases in the world. Insulin resistance in T2DM is associated with both hyperglycemia and hyperlipidemia, which are associated with an imbalance between endocrine pancreatic function and hepatic and extrahepatic insulin sensitivity. In recent years, traditional Chinese medicines and extracts have had unique advantages in the treatment of T2DM. Modern pharmacological studies have also confirmed that *Rosa roxburghii* Tratt has potential antidiabetic effects, but its material basis and mechanism of action remain to be identified. Therefore, using bioinformatics analysis including network pharmacology and molecular docking are needed to explore the molecular mechanism of *Rosa roxburghii* Tratt fruit in the treatment of T2DM. It is beneficial to explain the modern scientific basis of *Rosa roxburghii* Tratt fruit in the treatment of T2DM.

By constructing the compound-target network, we found that all 20 active compounds had interactive effects with multiple targets, indicating that *Rosa roxburghii* Tratt fruit has a strong pertinence in treating T2DM. In addition, many of these compounds had common targets, suggesting that different compounds may provide synergistic effects. The identified compounds were mainly triterpenoids and flavonoids. The flavonoids of *Rosa roxburghii* Tratt can significantly reduce serum glucose and triglyceride and increase serum insulin in experimental hyperglycemic mice induced by alloxan. The extract increased superoxide dismutase (SOD) and catalase (CAT) activities and decreased malondialdehyde (MDA) [39]. Furthermore, the extract of triterpenoids, flavonoids, and polysaccharides of *Rosa roxburghii* Tratt had favorable inhibitory effects against *α*-glucosidase activities [40, 41]. Polyphenols of *Rosa roxburghii* Tratt exhibit good hypoglycemic effects by activating the P13K/AKT signaling pathway and regulating the expression of the Forkhead box protein O1 (FOXO1), and glycogen synthase kinase-3 beta (GSK-3β) proteins, controlling liver gluconeogenesis and improving insulin resistance to hepatic glycogen storage, to alleviate symptoms of T2DM [42].

Thirteen targets, including AKT1, TNF, SRC, VEGFA, EGFR, CASP3, MAPK3, STAT3, PPARG, ESR1, MTOR, PTGS2, and MAPK1, were proposed as the possible key targets of *Rosa roxburghii* Tratt fruit for T2DM treatment. Among the top six core genes, the first AKT1 is one of the three members of the AKT family, AKT1/PKB*α*, AKT2/PKB*β,* and AKT3/PKB*γ* [43]*.* AKT1 is involved in many processes, including metabolism, proliferation, cell activity, growth, and angiogenesis, and is responsible for regulating the body's glucose uptake by mediating insulin-induced transport of the SLC2A4/GLUT4 glucose transporter to the cell surface and plays an important role in maintaining blood sugar stability [44]. Tumor necrosis factor (TNF) is a macrophage secreted cytokine and can bind to the tumor necrosis factor receptor superfamily (TNFR) to directly induce inflammation. TNF can inhibit insulin-induced tyrosine phosphorylation of insulin receptor substrate 1 (IRS1) and glucose uptake, and promote degradation of the GKAP42 protein in adipocytes, thus causing T2DM and insulin resistance [45].

Steroid receptor coactivator (SRC) is a nonreceptor tyrosine kinase that is associated with the cell membrane and plays a key role in various signal transduction pathways. Inhibition of SRC (c-Src) activation was found to decrease endogenous ROS production and increase ATP production in diabetic mice with hyperlipidemia [46]. Vascular endothelial growth factor A (VEGFA) is a growth factor that plays a vital role in angiogenesis and endothelial cell growth. Hyperglycemia results in the overexpression of VEGFA, which is a critical factor in diabetic complications such as diabetic retinopathy and diabetic nephropathy [47].

The last two of the first six key genes are EGFR and CASP3. EGFR is a transmembrane tyrosine kinase receptor belonging to the ErbB family [48]. The epidermal growth factor receptor (EGFR) tyrosine kinase inhibitor decreased obesity in a murine model of leptin receptor-deficient T2DM. In a patient with diabetes lung cancer, treatment with an EGFR tyrosine kinase inhibitor led to improved glucose control, reduced obesity, and the development of insulin resistance [49]. CASP3 is one of the most critical enzymes in the apoptotic pathway, which is closely related to the occurrence of cancer, aging, and cardiovascular disease. CASP3 has been confirmed to be associated with apoptosis of pancreatic islet *β*-cell apoptosis, and apoptosis of *β*-cells can lead to decreased insulin secretion, which in turn leads to the appearance of T2DM [50].

In addition, the enrichment analysis of the KEGG pathway, the results showed that *Rosa roxburghii* Tratt fruit mainly regulates signaling pathways including the PI3K/AKT signaling pathway, the RAS signaling pathway and the AGE-RAGE signaling pathway for the treatment of T2DM. The PI3K/AKT signaling pathway is one of the key pathways for insulin to regulate blood glucose levels. The phosphorylation of insulin receptors leads to the activation of PI3K, subsequently recruiting signaling proteins that include AKT and its downstream mediators. All are critical steps for stimulating insulin-induced glucose transport [51]. In the RAS signaling pathway, the insulin receptor binds to the Shc substrate and then GRB2-SOS to activate RAS. Activated RAS can bind to and activate Raf-1, which in turn reactivates MAPKK/MEK, and then MEK activates MAPK/ERK. MAPK can directly phosphorylate transcription factors or activate other protein kinases to regulate insulin gene expression [52].

Molecular docking was further applied to verify the interaction between compounds and targets. The combination with the lower binding energy scores is more stable, and the binding energy ≤  − 5.0 kcal/mol was defined as the standard of well binding between ligands and receptors in some studies. As shown in Fig. 9, the control drug glyburide showed good binding activity for all receptors, which proves the reliability of the protein target, and a few compounds had values higher than -5 kcal/mol. Considering “EGFR- melianodiol” (Fig. 11) as an example, the binding affinity was − 9.15 kcal/mol and was stabilized by five H-bonds with residues that included two glutamine amino acid residues (GLN-47 and GLN-88), a serine amino acid residue (SER-81), an isoleucine amino acid residue (ILE-83), and an arginine residue (ARG-131).

However, there are some limitations in this work. Less references and databases were collected to predict the active components and potential mechanisms of *Rosa roxburghii* Tratt fruit, so we use mass spectrometry to make up for the total amount of selected and evaluated compounds. Because the components of *Rosa roxburghii* Tratt are not clear and commercial, it is difficult to carry out experimental verification temporarily. At present, there are many varieties and origins of *Rosa roxburghii* Tratt with unclear quality markers and uneven quality, so we will try our best to establish quality standards for *Rosa roxburghii* Tratt in the future*.*

## Conclusions

This study explored the active components and potential mechanisms of *Rosa roxburghii* Tratt fruit against T2DM by UPLC-Q-Exactive Orbitrap/MS and network pharmacology. We finally obtained 20 key compounds and identified 214 potential targets active against T2DM, among which AKT1, TNF, SRC, VEGFA, EGFR, CASP3, MAPK3, STAT3, PPARG, ESR1, MTOR, PTGS2, and MAPK1 were the key targets. The mechanism of *Rosa roxburghii* Tratt fruit treatment of T2DM involves a complex network with multiple targets and pathways. *Rosa roxburghii* Tratt fruit can play a therapeutic role in T2DM by regulating pathways including the PI3K/AKT signaling pathway, the RAS signaling pathway, the AGE-RAGE signaling pathway and resistance to the EGFR tyrosine kinase inhibitor. Although the present results are found to be consistent with some reports, indicating the rationality and accuracy of our method, further experiments in vivo and in vitro are still needed to validate these predictions. At present, most research are performed to explore the function of fruit from *Rosa roxburghii* Tratt, as well as the product development of *Rosa roxburghii* Tratt mainly is focused on fruit juice, but there are few novel products to be developed in other product forms as functional food and dietary supplements. There are less academic research and R&D development on the bioactive ingredients of *Rosa roxburghii* Tratt, so the work are still needed to perform in-depth and systematic research and promote the further development and utilization of these edible and medicinal resources in future.

## Supplementary Information

**Additional file 1: Table S1.** Chromatographic and MS/MS spectral information for the constituents detected in the QC sample of *Rosa roxburghii* Tratt fruit using UPLC-Q-Exactive Orbitrap/MS. **Figure S1.** The MS^2^ spectrum of compound **58**. **Figure S2.** Proposed mass fragmentation pathways in response to the primary fragment ion species of a representative triterpenoid Alphitolic acid. **Figure S3.** The MS^2^ spectrum of compound **23**. **Figure S4.** Proposed mass fragmentation pathways in response to the primary fragment ion species of a representative flavonoid namely Catechin.

## Data Availability

The datasets used and analyzed during the current study are available from the corresponding author upon reasonable request.

## Abbreviations

ADMET: Absorptions, distribution, metabolism, excretion, and toxicity
AKT1: Serine/threonine kinase 1
BC: Betweenness centrality
BP: Biological process
CASP3: Caspase-3
CAT: Catalase
CC: Closeness centrality
DC: Degree centrality
DL: Drug-likeness
EGFR: Epidermal growth factor receptor
ESR1: Estrogen receptor 1
FOXO1: Forkhead box protein O1
GO: Gene ontology
GSK-3β: Glycogen synthase kinase-3 beta
IRS1: Insulin receptor substrate 1
KEGG: Kyoto encyclopedia of genes and genomes
mTOR: Mammalian target of rapamycin
MAPK1: Mitogen-activated protein kinase 1
MDA: Malondialdehyde
MAPK3: Mitogen-activated protein 3
MF: Molecular function
OB: Oral bioavailability
PI3K: Phosphatidylinositol 3-kinase
STAT3: Signal transducer and activator of transcription 3
SOD: Superoxide dismutase
SRC: Steroid receptor coactivator
PTGS2: Prostaglandin-endoperoxide synthase 2
PPARG: Peroxisome proliferator-activated receptor gamma
T2DM: Type 2 diabetes mellitus
TCMSP: Traditional Chinese medicine system pharmacology analysis platform
TNF: Tumor necrosis factor
TNFRs: Tumor necrosis factor receptor superfamily
VEGFA: Vascular endothelial growth factor A
