# Supplementary material for: Exploring the active components and potential mechanisms of Rosa roxburghii Tratt in treating type 2 diabetes mellitus based on UPLC-Q-exactive Orbitrap/MS and network pharmacology
Source: Chin Med. 2023 Feb 6;18:12. doi: 10.1186/s13020-023-00713-z (PMC9903504; doi:10.1186/s13020-023-00713-z)
Supplement: Supplementary file 1 — Additional file 1: Table S1. Chromatographic and MS/MS spectral information for the constituents detected in the QC sample of Rosa roxburghii Tratt fruit using UPLC-Q-Exactive Orbitrap/MS. Figure S1. The MS2 spectrum of compound 58. Figure S2. Proposed mass fragmentation pathways in response to the primary fragment ion species of a representative triterpenoid Alphitolic acid. Figure S3. The MS2 spectrum of compound 23. Figure S4. Proposed mass fragmentation pathways in response to the primary fragment ion species of a representative flavonoid namely Catechin. [file 13020_2023_713_MOESM1_ESM.docx]

**Exploring the active components and potential mechanisms of *Rosa roxburghii* Tratt in treating Type 2 Diabetes Mellitus based on** **UPLC-Q-Exactive Orbitrap/MS and network pharmacology**

Chenxiao Shen^1†^, Yu Wang^2†^, Hui Zhang^2^, Wei Li ^3^, Wenyue Chen^1^, Mingqing Kuang^2^, Yuelin Song^3*^ and Zhangfeng Zhong^1*^

1. Macao Centre for Research and Development in Chinese Medicine, Institute of Chinese Medical Sciences, University of Macau, Macao SAR 999078, China.
2. Guangzhou Wanglaoji Health Industry Co, Ltd, Guangzhou 510632, China.
3. Modern Research Center for Traditional Chinese Medicine, School of Chinese Materia Medica, Beijing University of Chinese Medicine, Beijing 100029, China.

*Correspondence: zfzhong@um.edu.mo (ZFZ); syltwc2005@163.com (YLS)

| **Table S1** Chromatographic and MS/MS spectral information identities for the constituents detected in the QC sample of *Rosa roxburghii* Tratt fruit using UPLC-Q-Exactive Orbitrap/MS | | | | | | | | | | |
| --- | --- | --- | --- | --- | --- | --- | --- | --- | --- | --- |
| No. | *t*_R_ (min) | Adducts | MS^1^ (*m/z*) | Ion mode | Error  (ppm) | MS^2^ (*m/z*) | Molecular formula | Identity | Chemical class | Ref. |
| **1** | 0.98 | [M+H]^+^ | 151.0378 | Pos | −7.75 | 123.0142,105.0036 | C_8_H_6_O_3_ | 3-Formylbenzoic acid | Organic acid | [1] |
| **2** | 1.08 | [M+H]^+^ | 147.1128 | Pos | −0.28 | 84.081 | C_6_H_14_N_2_O_2_ | Lysine | Amino acid | [2] |
| **3** | 1.18 | [M+H]^+^ | 175.1189 | Pos | −0.32 | 116.0710,70.0654,60.0570 | C_6_H_14_N_4_O_2_ | Arginine | Amino acid | [2,3] |
| **4** | 1.25 | [M−H]^−^ | 191.0567 | Neg | 9.14 | 85.0289 | C_7_H_12_O_6_ | Quinic acid | Organic acid | [2-4] |
| **5** | 1.35 | [M−H]^−^ | 133.0148 | Neg | 13.22 | 115.0043,71.0139 | C_4_H_6_O_5_ | Malic acid | Organic acid | [2-4] |
| **6** | 1.76 | [M−H]^−^ | 175.0255 | Neg | 10.10 | 115.0044,87.0090,71.0139.59.0138 | C_6_H_8_O_6_ | Ascorbic acid | Organic acid | [2,3,5] |
| **7** | 1.8 | [M+H]^+^ | 132.1018 | Pos | −0.75 | 86.0966,69.0702,56.0499 | C_6_H_13_NO_2_ | Leucine | Amino acid | [2,3] |
| **8** | 1.92 | [M−H]^−^ | 191.0205 | Neg | 9.59 | 111.0092 | C_6_H_8_O_7_ | Citric acid | Organic acid | [2,3,5] |
| **9** | 1.97 | [M−H]^−^ | 481.0627 | Neg | 2.91 | 300.9993,275.0194,257.0093 | C_20_H_18_O_14_ | 2,3-HHDP-glucose/4,6-HHDP-glucose | Tannins | [6] |
| **10** | 2.28 | [M−H]^−^ | 331.0678 | Neg | 5.55 | 271.0454,211.0253,169.0150,151.0044 | C_13_H_16_O_10_ | *β*-glucogallin | Flavonoids | [7] |
| **11** | 2.49 | [M−H]^−^ | 343.0682 | Neg | 6.60 | 191.0567,125.0254 | C_14_H_16_O_10_ | 3-Galloylquinic acid | Organic acid | [8] |
| **12** | 2.92 | [M+H]^+^ | 169.015 | Neg | 11.03 | 125.0251 | C_7_H_6_O_5_ | Gallic acid | Organic acid | [2,3] |
| **13** | 4.75 | [M−H]^−^ | 289.0929 | Neg | 3.69 | 127.0407,113.0251,101.0249 | C_12_H_18_O_8_ | Dianthoside | Triterpenoids | [9] |
| **14** | 5.03 | [M−H]^−^ | 305.067 | Neg | 4.82 | 167.0356,125.0252 | C_15_H_14_O_7_ | Gallocatechin | Flavonoids | [2,3] |
| **15** | 5.45 | [M−H]^−^ | 481.0991 | Neg | 2.96 | 289.0717,245.0823 | C_21_H_22_O_13_ | Epigallocatechin 3-glucuronide | Flavonoids | −− |
| **16** | 5.5 | [M−H]^−^ | 153.0201 | Neg | 12.06 | 109.0300,91.0190,81.0347 | C_7_H_6_O_4_ | Protocatechuic acid | Phenolic acids | [2,3] |
| **17** | 5.55 | [M−H]^−^ | 463.088 | Neg | 2.03 | 445.0787,327.0515,205.0147,177.02 | C_21_H_20_O_12_ | Unknown | − | − |
| **18** | 6.64 | [M−H]^−^ | 463.0883 | Neg | 2.69 | 373.0575,327.0515,309.0405,205.0151,177.0200,135.0305 | C_21_H_20_O_12_ | Unknown | − | − |
| **19** | 7.46 | [M−H]^−^ | 271.0821 | Neg | 3.30 | 109.03 | C_12_H_16_O_7_ | Arbutin | Flavonoids | [10] |
| **20** | 8.27 | [M−H]^−^ | 577.1373 | Neg | 5.68 | 451.0782,425.0882,407.0782,289.0717,245.0821,161.0252,125.0250 | C_30_H_26_O_12_ | Proanthocyanidin B1 | Flavonoids | [2,4] |
| **21** | 9.19 | [M−H]^−^ | 577.1375 | Neg | 5.89 | 451.1035,425.0884,407.0780,289.0717,161.0251,125.0250 | C_30_H_26_O_12_ | Proanthocyanidin B3 | Flavonoids | [2,3] |
| **22** | 9.36 | [M−H]^−^ | 463.0883 | Neg | 2.49 | 327.0516,175.0255,135.0306,115.0042,87.0090 | C_21_H_20_O_12_ | Unknown | − | − |
| **23** | 10.13 | [M−H]^−^ | 289.0718 | Neg | 4.07 | 271.0602,245.0825,203.0581,163.0403,137.0251,123.0458,109.0300 | C_15_H_14_O_6_ | Catechin* | Flavonoids | [3,4] |
| **24** | 10.49 | [M−H]^−^ | 325.0935 | Neg | 5.27 | 163.0408,145.0302,119.0507 | C_15_H_18_O_8_ | Melilotoside | Flavonoids | [11] |
| **25** | 10.53 | [M+H]^+^ | 147.044 | Pos | −0.55 | 119.0495,91.0545 | C_9_H_6_O_2_ | Coumarin | Coumarins | [3] |
| **26** | 11.3 | [M−H]^−^ | 633.0793 | Neg | 10.65 | 300.9993,275.0090 | C_27_H_22_O_18_ | Corilagin | Tannins | [12] |
| **27** | 13.11 | [M−H]^−^ | 431.1931 | Neg | 4.50 | 385.1880,223.1329,205.1240 | C_19_H_30_O_8_ | Sonchuionoside C | Flavonoids | −− |
| **28** | 13.47 | [M−H]^−^ | 437.1099 | Neg | 4.77 | 289.0718,245.0822 | C_20_H_22_O_11_ | Loquatoside | Flavonoids | −− |
| **29** | 13.9 | [M−H]^−^ | 577.1373 | Neg | 5.68 | 425.0882,407.0782,289.0717,245.0822,203.0723,161.0251,125.0250 | C_30_H_26_O_12_ | Proanthocyanidin B2* | Flavonoids | [3,4] |
| **30** | 15.26 | [M−H]^−^ | 729.1536 | Neg | 3.67 | 577.1378,407.0783.289.0717 | C_30_H_34_O_21_ | Unknown | − | − |
| **31** | 15.36 | [M−H]^−^ | 577.1373 | Neg | 5.57 | 407.0779,289.0716,245.0821,203.0718,161.0250,125.0250 | C_30_H_26_O_12_ | Proanthocyanidin B4 | Flavonoids | −− |
| **32** | 16.13 | [M−H]^−^ | 433.0422 | Neg | 5.03 | 299.9914,283.9965,229.0154 | C_19_H_14_O_12_ | Ellagic acid 4-O-xylopyranoside | Tannins | −− |
| **33** | 16.48 | [M−H]^−^ | 447.0534 | Neg | 3.44 | 299.9915 | C_20_H_16_O_12_ | Ellagic acid deoxyhexoside | Tannins | −− |
| **34** | 16.72 | [M−H]^−^ | 137.0251 | Neg | 13.13 | 93.0349,75.0242,65.0397 | C_7_H_6_O_3_ | Salicylic acid | Phenolic acids | [13] |
| **35** | 17.03 | [M−H]^−^ | 300.9992 | Neg | 4.47 | 283.9965,257.0090,229.0146 | C_14_H_6_O_8_ | Ellagic acid | Tannins | [3,13] |
| **36** | 17.2 | [M−H]^−^ | 463.0883 | Neg | 2.49 | 300.0278,271.0245,255.0299 | C_21_H_20_O_12_ | Isoquercitrin | Flavonoids | [2,3,13] |
| **37** | 17.23 | [M+H]^+^ | 465.1023 | Pos | −1.06 | 303.0501,257.0448,153.0185,127.0393 | C_21_H_20_O_12_ | Hyperoside | Flavonoids | [3,13] |
| **38** | 17.75 | [M−H]^−^ | 609.1359 | Neg | −14.68 | 300.0277,255.0297,151.0043 | C_27_H_30_O_16_ | Rutin | Flavonoids | [2,3,13] |
| **39** | 17.79 | [M−H]^−^ | 607.1317 | Neg | 3.87 | 505.0978,463.0884,300.0279 | C_27_H_28_O_16_ | 6''-(4-Carboxy-3-hydroxy-3-methylbutanoyl) hyperin | Flavonoids | −− |
| **40** | 18.1 | [M−H]^−^ | 579.2108 | Neg | 6.10 | 417.1562,387.1092,181.0513,166.0278 | C_28_H_36_O_13_ | Acanthoside B | Flavonoids | −− |
| **41** | 19.64 | [M+HCOO] ^−^ | 711.4047 | Neg | −2.15 | 665.3946,503.3369,407.2965,207.0517 | C_36_H_58_O_11_ | Niga-ichigoside F1/isomer | Triterpenoids | −− |
| **42** | 19.74 | [M+HCOO] ^−^ | 711.4041 | Neg | 12.96 | 665.3948,503.3368,407.2964 | C_36_H_58_O_11_ | Niga-ichigoside F1/isomer | Triterpenoids | −− |
| **43** | 20.84 | [M+HCOO] ^−^ | 711.4046 | Neg | −2.32 | 665.3957,503.3371,207.0516 | C_36_H_58_O_11_ | Niga-ichigoside F1/isomer | Triterpenoids | −− |
| **44** | 20.94 | [M+HCOO] ^−^ | 693.3943 | Neg | 14.27 | 647.3850,485.3273,207.0520 | C_37_H_58_O_12_ | Oligoporin C/Acanthosessilioside D/quadranoside VIII | Triterpenoids | −− |
| **45** | 23.84 | [M−H]^−^ | 649.3986 | Neg | 6.25 | 649.4000,487.3427,469.3319,425.3420,407.3354,207.0520 | C_36_H_58_O_10_ | Kajiichigoside F1/isomer | Triterpenoids | [3,4] |
| **46** | 23.86 | [M−H]^−^ | 487.3426 | Neg | 1.56 | 469.3299 | C_30_H_48_O_5_ | Melianodiol | Triterpenoids | [14] |
| **47** | 24.5 | [M+HCOO] ^−^ | 695.4103 | Neg | 14.66 | 649.4002,487.3426,207.0516 | C_36_H_58_O_10_ | Kajiichigoside F1/isomer | Triterpenoids | [2,3] |
| **48** | 25.2 | [M+HCOO] ^−^ | 695.4102 | Neg | 14.63 | 649.4005,487.3425,427.3232,207.0515 | C_36_H_58_O_10_ | Kajiichigoside F1/isomer | Triterpenoids | [2,3] |
| **49** | 25.64 | [M+HCOO] ^−^ | 693.3947 | Neg | 14.79 | 647.3846,485.3271,207.0515 | C_37_H_58_O_12_ | Oligoporin C/Acanthosessilioside D/quadranoside VIII | Triterpenoids | −− |
| **50** | 25.85 | [M+HCOO] ^−^ | 693.3948 | Neg | 14.98 | 647.3850,485.3271,207.0515 | C_37_H_58_O_12_ | Oligoporin C/Acanthosessilioside D/quadranoside VIII | Triterpenoids | −− |
| **51** | 33.43 | [M−H]^−^ | 487.3419 | Neg | 0.24 | 469.3324,443.3499 | C_30_H_48_O_5_ | Euscaphic acid | Triterpenoids | [2,3] |
| **52** | 33.57 | [M−H]^−^ | 503.3369 | Neg | 0.27 | 485.3268,441.3336,421.3118 | C_30_H_48_O_6_ | Roxburic acid/1*α*,2*β*,3*β*,19*α*-tetrahydroyurs-12-en-28-oic acid | Triterpenoids | [15] |
| **53** | 33.74 | [M−H]^−^ | 485.327 | Neg | 1.80 | 467.3172,441.3387,405.3185 | C_30_H_46_O_5_ | 2-oxo-pomoloc acid/2*α*,19*α*-dihydroxy-3-oxo-urs-12-en-28-oic acid | Triterpenoids | [2] |
| **54** | 33.95 | [M−H]^−^ | 487.3429 | Neg | 2.32 | 469.3330,423.3291, | C_30_H_48_O_5_ | Tormentic acid | Triterpenoids | [3] |
| **55** | 33.96 | [M−H]^−^ | 485.3271 | Neg | 1.87 | 467.3174,423.3289 | C_30_H_46_O_5_ | Ceanothic acid | Triterpenoids | [13] |
| **56** | 34.15 | [M−H]^−^ | 501.3214 | Neg | 0.63 | 483.3120,465.3012,421.3123,377.2864 | C_30_H_46_O_6_ | Medicagenic acid/isomer | Triterpenoids | −− |
| **57** | 34.47 | [M−H]^−^ | 501.3211 | Neg | 1.05 | 483.3118,465.3017,421.3120 | C_30_H_46_O_6_ | Medicagenic acid/isomer | Triterpenoids | −− |
| **58** | 34.87 | [M−H]^−^ | 471.3485 | Neg | 3.57 | 453.3365,427.3548 | C_30_H_48_O_4_ | Alphitolic acid | Triterpenoids | [2] |

Note：“*” identified by authentic reference.

“—”, identified through database.


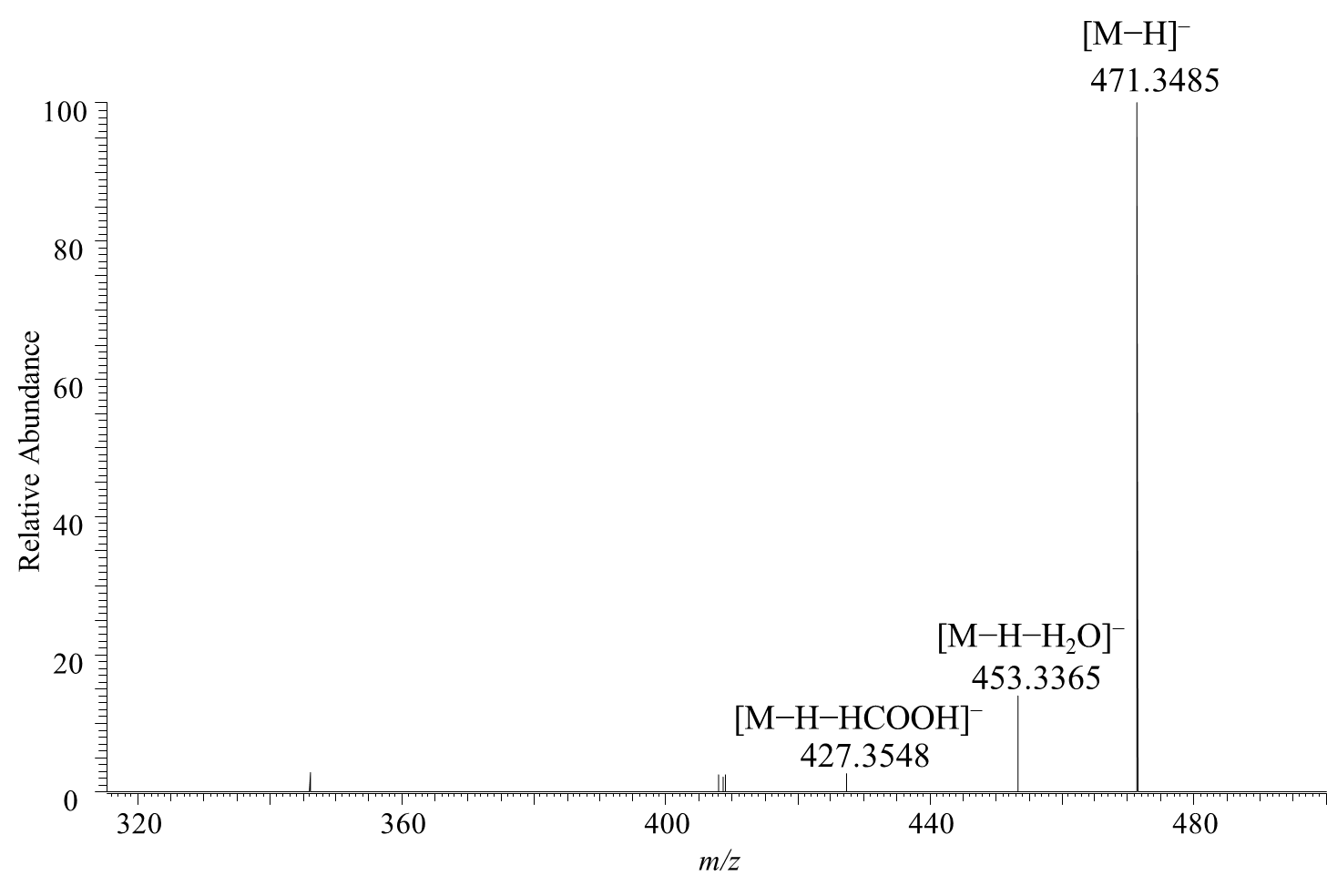


**Fig. S1** The MS^2^ spectrum of compound **58**.

**
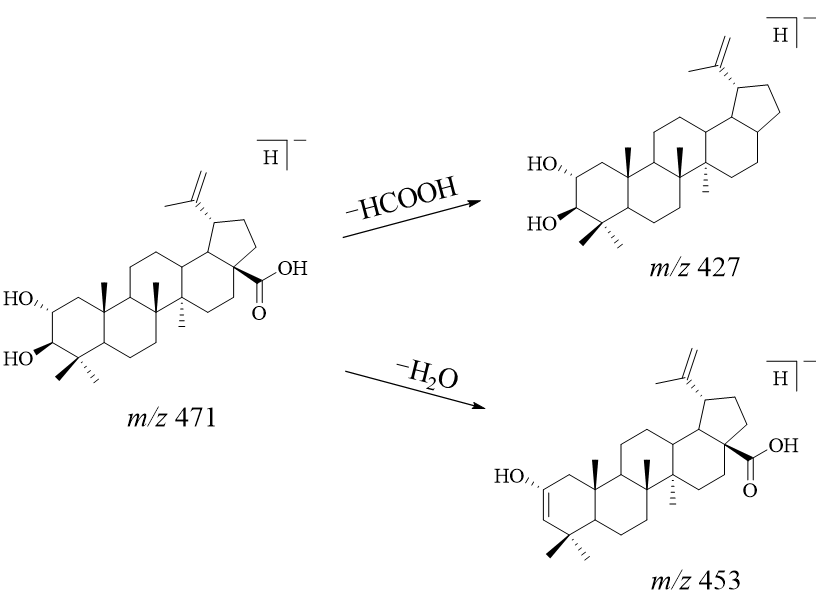
**

**Fig. S2** Proposed mass fragmentation pathways in response to the primary fragment ion species of a representative triterpenoids namely Alphitolic acid.


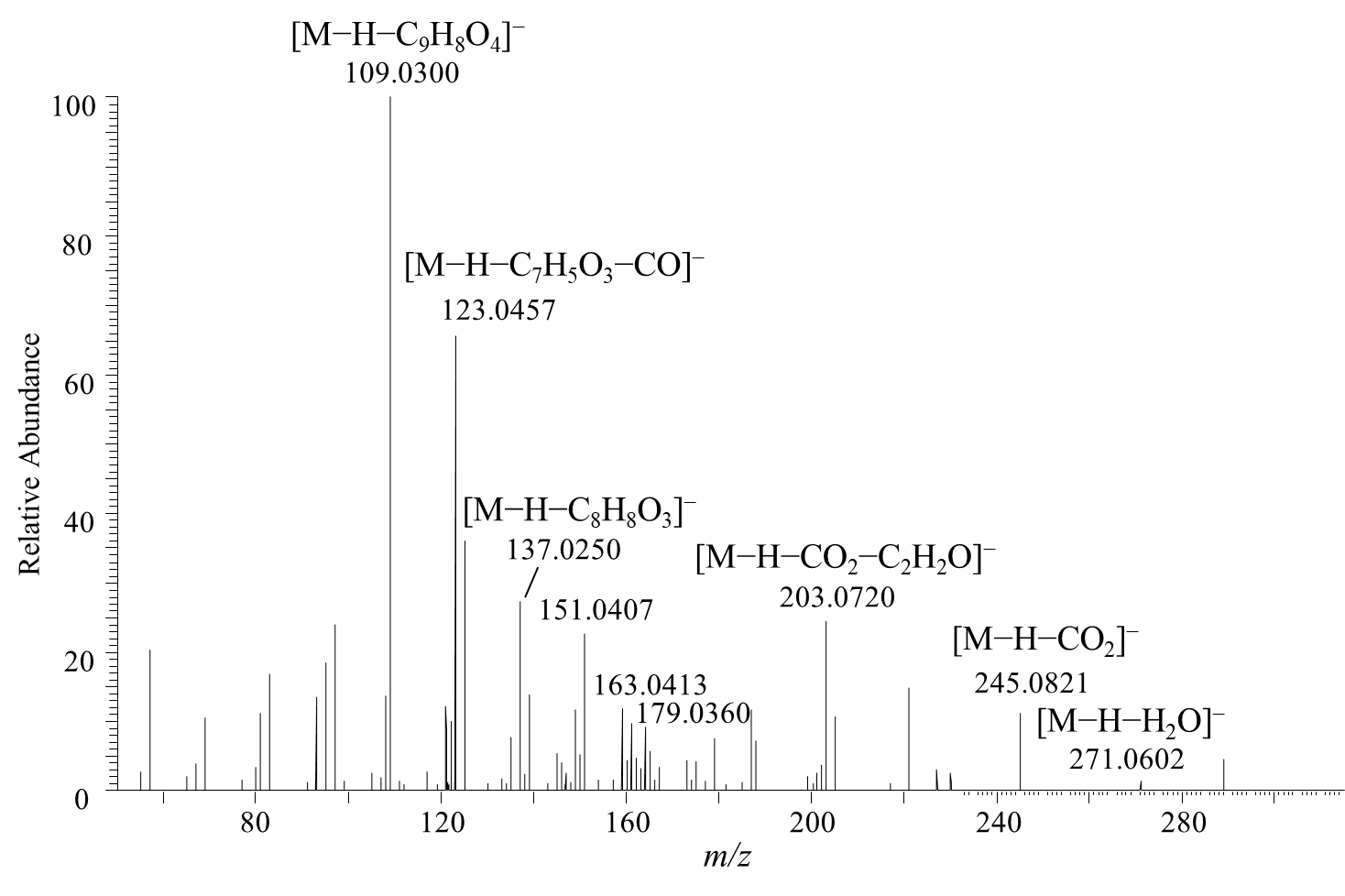


**Fig. S3** The MS^2^ spectrum of compound **23**.

**Fig. S4** Proposed mass fragmentation pathways in response to the primary fragment ion species of a representative flavonoids namely Catechin.

**Reference：**

1. Yuan JJ. Enzymatic hydrolysis mechanism of oleuropein extract and activity structure-activity relationship of phenolic products. Chinese Academy of Forestry Sciences. 2016; 41-41.
2. Liu M, Zhang Q, Zhang Y, Lu X, Fu W and He J. Chemical analysis of dietary constituents in Rosa roxburghii and Rosa sterilis Fruits. Molecules. 2016; 21: 1204. <https://doi.org/10.3390/molecules21091204>.
3. Xu HR, Qiao XY, Zhang TH, Yu GY, Bi YL, Feng X, Wei KY, Sun YK. Analysis of chemical components of miao medicine Rosae Roxburghii Folium Based on UPLC-Q-Exactive-Orbitrap−MS[J/OL]. Mod Chin Med. 2022; 1-12.
4. Zeng FF, Ge ZW, Jarukitt L, Li L, Feng SM, Wang YS, Luo ZS. Antioxidant and tyrosinase inhibitory activity of Rosa roxburghii fruit and identification of main bioactive phytochemicals by UPLC-Triple-TOF/MS. Food Sci Tech. 2017; 52: 897-905. <https://doi.org/10.1111/ijfs.13353>.
5. He JY, Zhang YH, Ma N, Liu MH, Fu WM. Comparative analysis of multiple ingredients in Rosa roxburghii and R. sterilis fruits and their antioxidant activities. J Funct Foods. 2016; 27:29-41. <https://doi.org/10.1016/j.jff.2016.08.058>.
6. Wei YF. Component Analysis and Antitumor and Antioxidant effects of Rubus corchorifolius L.f. roots. Guangxi University of Traditional Chinese Medicine. 2018; 20-21.
7. Tong Z, wen QY, Ping W, Nengwu Z. Screening and mechanism analysis of active components of Moutan Cortex in treatment of chronic nephritis based on UHPLC-QTOF-MS/MS and TCMIP. Chinese Medicinal Materials. 2022; 53(9):2756-2767.
8. Yan HF, Wang ML, Zou C, Chen L, Liu XY, Dai JY. Determination of Water–Soluble Compounds in Black Tea by LC–IT–TOF MS Method. Chemical analysis and metering. 2017; 26(03):22-26.
9. Huang YL, Zhang YL, Xiang Q, Yu R, Wu YJ, Liu X. Rapid analysis of chemical components in Zuogui Jiangtang Shuxin Formular by UHPLC - Q - Exactive - Orbitrap Mass Spectrometry. Instrumental Analysis. 2022; 47(07):963-977.
10. Ren H, Cui XM, Hu J, Liu XM, Chen ZY, Zhang QY. Analysis on Chemical Constituents in Rhizomes of Bergenia scopulosa by UHPLC-Q Exactive Focus MS/MS. Chinese Journal of Experimental Traditional Medical Formular. 2021; 27(9):118-128.
11. Ma BJ, Xiao Y, Chen ZD, Shu RG, Li BT, Jiang L, Xu GL, Zhang QY. Analysis of Chemical Constituents in Percolate the Extract of Cyclocarya paliurus Tender Leaves by UHPLC-Q-TOF-MS/MS. Science and Technology of Food Industry. 2022; 1-17.
12. Liu CY, Dai YP, Wang BL, Ding XY, Jiang HQ, Zhou Q. Rapid Analysis of Tannins from Pomegranate Peel Based on UPLC-Quadrupole/Exactive Orbitrap Mass Combined with Diagnostic Ions Filter. Chinese Archives of Traditional Chinese Medicine. 2022; 1-18.
13. Jiang LL, Lu M, An HM. Comparative analysis of chemical components of traditional Chinese medicine and antioxidants in Rosa roxburghii and Rosa sterilis fruits [J/OL]. J Fruit Science.2022; 1-27.
14. Kenneth OE, Harquin SF, Guerisson B, Gabriel NF, Emanuel AA, Andreas L, Marc L. A new ursane triterpenoic acid and other potential anti-inflammatory and anti-arthritic constituents from EtOAc extracts of Vitellaria paradoxa stem bark, J Ethnopharmacol, 2015; 174, 277-286. <https://doi.org/10.1016/j.jep.2015.08.014>.
15. Yang Q, Zhang D, Farha A K. Phytochemicals, essential oils, and bioactivities of an underutilized wild fruit Cili (Rosa roxburghii). Ind. Crops Prod. 2020; 143:111928. https://doi.org/10.1016/j.indcrop.2019.111928.
